# Assessing microbiome engraftment extent following fecal microbiota transplant with q2-fmt

**DOI:** 10.1371/journal.pcbi.1013299

**Published:** 2025-07-31

**Authors:** Chloe Herman, Evan Bolyen, Anthony Simard, Liz Gehret, J. Gregory Caporaso

**Affiliations:** 1 Pathogen and Microbiome Institute, Northern Arizona University, Flagstaff, Arizona, United States of America; 2 School of Informatics, Computing and Cyber Systems, Northern Arizona University, Flagstaff, Arizona, United States of America; Clemson University, UNITED STATES OF AMERICA

## Abstract

We present q2-fmt, a QIIME 2 plugin that provides diverse methods for assessing the extent of microbiome engraftment following fecal microbiota transplant. The methods implemented here were informed by a recent literature review on approaches for assessing FMT engraftment, and cover aspects of engraftment including Community Coalescence, Indicator Features, and Resilience. q2-fmt is free for all use, and detailed documentation illustrating worked examples on a real-world data set are provided in the project’s documentation.

## Author summary

Fecal Microbiota Transplants (FMTs) are a treatment for recurrent *Clostridium difficile* infections and are being investigated as treatments for many other conditions. To determine if an FMT was successful, we need to assess how the microbiome of a donor is transferred into a recipient following FMT. q2-fmt is software for quantifying this transfer with diverse methods incorporated in one of the most widely used microbiome analysis platforms, QIIME 2. This allows q2-fmt users to investigate their FMT data in the microbiome platform that they are already using for their microbiome analysis and get all the benefits of working in QIIME 2, including full analysis reproducibility through retrospective provenance tracking, access to the same tools through interfaces targeting users with differing computational backgrounds, and integration with other widely used microbiome analysis tools.

## Introduction

Methods for assessing microbial engraftment following Fecal Microbiota Transplants (FMTs) are not standardized, making it difficult for researchers to investigate if FMT was an effective treatment for their condition of interest. Quantifying engraftment extent after FMTs is pivotal to interpreting the results of an FMT study. For example, if no positive clinical outcome is achieved, is it because FMT is not a relevant treatment option for the condition, or because the microbiome did not successfully engraft?

q2-fmt is a QIIME 2 plugin for assessing microbiome engraftment extent following FMT. Accessibility of this functionality through QIIME 2 provides an all-in-one suite for assessing FMT engraftment in a software platform that many microbiome researchers are already familiar with.

Herman et al. 2024 [1] defined a framework for assessing engraftment with three core concepts for assessing engraftment extent. These three concepts are *Community Coalescence*, *Indicator Features*, and *Resilience*. *Community Coalescence* quantifies the extent to which a recipient’s microbiome composition shifts towards a donated microbiome following FMT. *Indicator Features* tracks specific microbiome features that are present in the donated microbiome (e.g., specific amplicon sequence variants, or a genus of interest) in the recipient’s microbiome following FMT. *Resilience* tracks the extent to which the microbiome maintains similarity to the donated microbiome following FMT. q2-fmt provides functionality to address all three of these criteria.

## Design and implementation

### Community Coalescence

#### Community Coalescence (CC) Pipeline.

qiime fmt cc is a pipeline that generates “raincloud plots” and associated statistics tracking microbiome composition changes over time. qiime fmt cc operates on microbiome alpha or beta diversity metrics (QIIME 2 semantic types: SampleData[AlphaDiversity] or DistanceMatrix, respectively). If a beta diversity distance matrix is provided (as opposed to an alpha diversity metric), the user can select whether to investigate the microbiome composition distance between the recipient and either the donor or the recipient’s baseline. The cc pipeline does this by running three independent actions.

**Fig 1:**
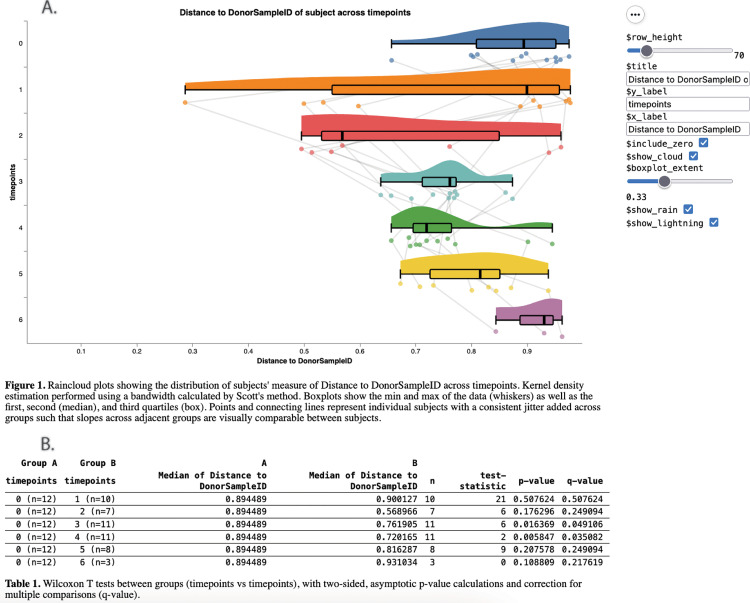
Jaccard Distance to Donor following FMT intervention, as assessed using qiime fmt cc. This visualization allows for users to interactively investigate whether recipient microbiomes become more similar to donor microbiomes with treatment (Fig 1A), and investigate statistical significance in the corresponding table (Fig 1B). In this figure, FMT intervention was at timepoint 3. Since this is an autoFMT study, the ‘donor’ is the recipient before cancer treatment. This figure shows that spontaneous recovery from cancer treatment leads to some individuals recovering quickly, while others do not. The FMT intervention decreases the variability in distance to donor of all recipients. We see that their distance to donor increases at the last time point, but this is up to two years after the initial FMT, and may indicate the development of an “individualized microbiome.” Presented data is from Taur *et al.* (2018) [2]. An interactive version of this figure is available in the project documentation at https://q2-fmt.readthedocs.io.

First, qiime fmt group-timepoints prepares diversity metrics for analysis by creating a table (QIIME 2 semantic type: Dist1D) that contains relevant metadata (subject identifier, timepoint identifier, donor identifier) and diversity metrics (alpha diversity values, or microbiome distances to per-subject baseline or donor samples). Second, relevant statistics are computed by the q2-stats plugin (typically Wilcoxon SRT; https://github.com/qiime2/q2-stats), and a statistics table (QIIME 2 semantic type: StatsTable[Pairwise]) is created. Lastly, the Dist1D and StatsTable are provided to qiime stats plot-raincould, generating a QIIME 2 visualization containing a raincloud plot and the associated statistics (Fig 1).

#### Proportional Engraftment of Donor Features (PEDF).

Proportional Engraftment of Donor Strains (PEDS) [3] was first defined by Aggarwala et al, 2020 and reports the proportion of donor features (e.g., amplicon sequence variants) that are observed in a recipient sample. We have generalized this methodology as Proportional Engraftment of Donor Features, PEDF, to enable investigation of any type of microbiome feature (e.g., OTUs, ASV, taxa, or metabolites). qiime fmt pedf uses microbiome feature tables (QIIME 2 semantic type: FeatureTable) as input and calculates the number of donor features observed in each recipient sample divided by the total number of donor features. In QIIME 2, this metric can be visualized using a heatmap by running qiime fmt heatmap, or using a raincloud plot (like that shown in Fig 1) to track how PEDF changes over time following an FMT. This method enables researchers to understand the proportion of donor features that were transferred to and persisted in the recipient over time.

**Fig 2:**
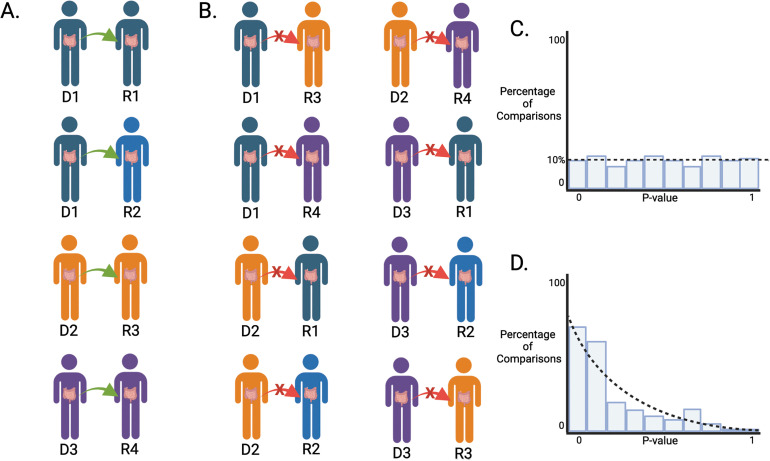
Schematic of PEDF permutation test. To test if PEDF is capturing donor overlap caused by microbiome transfer versus general microbiome similarity that could be observed across any two microbiomes in the study, we developed the PEDF permutation test. The PEDF permutation test breaks connections between factual donor-recipient pairs and tests to see if the unrelated donor-recipient pairs are more similar than the related donor-recipient pairs (via FMT transplant)**. A)** The combinations of actual donor-recipient pairs in this example. Note Donor 1 is a donor to two recipients, to illustrate how PEDF permutation handles repeated transfers from one donor. PEDF values would be calculated for each of these pairs, and these would be considered our factual PEDF values. **B)** To create a null (i.e., counterfactual) distribution to compare factual PEDF values against, donors are paired up with every recipient that they did not donate to, to form our counterfactual pairs. The counterfactual pairs are sampled 999 times, and PEDF is calculated for each counterfactual pair to provide a robust null distribution. If the recipients have significant donor similarity following the FMT, it is expected that our factual donor-recipient pairs will have higher PEDF than our counterfactual donor-recipient pairs. The fraction of counterfactual PEDF values that are greater than an individual factual donor-recipient pair PEDF value is the p-value representing the significance of the factual donor-recipient pair PEDF value. **C)** A distribution of factual donor-recipient pair PEDF p-values with no globally significant signal will be distributed uniformly. **D)** If factual donor-recipient PEDF p-values are clustered around significant values, this suggests global statistical significance. This figure is a schematic representation and all data is simulated. Created in BioRender.

To assess whether PEDF is identifying actual transfer from the donor to the recipient, as opposed to simply shared microbiome features between donor and recipient samples, q2-fmt introduces a novel permutation test through the qiime fmt pedf-permutation-test action. pedf-permutation-test begins by defining “counterfactual donor pairs,” where recipient samples are paired with donor samples from the study that do not represent their actual donor (Fig 2B). Counterfactual donor-recipient pairs are sampled with replacement from the input data, and for each pair the samples are rarified to a user-specified even sampling depth, and PEDF is computed for the recipient sample. The number of counterfactual pairs sampled is defined using the num_resamples parameter, which defaults to 999, and rarifying is performed after sampling such that if the same counterfactual pair is drawn twice, the samples will be rarified independently. Then, PEDF is computed for each factual donor-recipient pair (Fig 2A) and the number of counterfactual donor pairs that achieved a PEDF score greater than or equal to the factual pair plus one is divided by num_resamples plus one to provide a permutation test p-value for that PEDF value for that factual pair. After computing all factual donor-recipient pairs’ PEDF p-values, we test if our recipients were overall more similar to their donor than the counterfactual pairs using Stouffer’s Method.[4] When there is no signal of significance, p-values are expected to be uniformly distributed (Fig 2C). This uniform distribution serves as the null distribution for the PEDF permutation global test. Stouffer’s Method tests if factual PEDF p-values collectively deviate from the null and cluster around significant values (Fig 2D), and provide a measure of global significance (which can be achieved even if some p-values are not significant).

#### Proportion of Recipients with Donor Feature (PRDF).

We additionally define a new metric related to PEDF that we refer to as Proportion of Recipients with Donor Feature, PRDF. The prdf action takes the same inputs as pedf but quantifies which features are more successful at engrafting into recipients as the proportion of recipients that successfully engrafted a specific feature when it was present in their donor as:

qiime fmt prdf is similarly able to be visualized using a heatmap to track which features engraft the most over time. This allows users to investigate whether certain microbes are more or less frequently transferred, thus enabling the generation of hypotheses regarding microbes that may or may not be amenable to engraftment to a new host.

#### Proportion of Persistent Recipient Features (PPRF).

Proportion of Persistent Recipient Features, or PPRF, is a generalized method adapted from Aggarwala et al 2021’s Proportion of Persistent Recipient Strains [3] or PPRS. PPRF captures the amount of features that are persistent in the recipient following FMT. This method was calculated by the number of unique baseline (pre-FMT) features that are still present in the recipient following FMT divided by the total number of baseline features. qiime fmt pprf uses a feature table and a baseline timepoint to calculate the PPRF for each recipient sample. Similar to qiime fmt pedf this outputs a Dist1D and can be visualized using qiime fmt heatmap.

### Indicator features

#### ANCOMBC.

qiime composition ancombc can be used to identify indicator features, or features that are significantly over-represented in donated microbiome samples relative to baseline recipient samples. This enables investigation of features that are more abundant in the donor and can serve as indicator features. In order to facilitate the detection of donor indicator features, we present qiime fmt detect-donor-indicators. This pipeline takes a feature table, a metadata column with donor sample IDs (which most q2-fmt actions require), and a baseline time point. This pipeline then filters the feature table down to donors and recipient baseline time point samples and runs ANCOMBC, comparing the donors to the recipients at baseline.

Future directions for identifying indicator features will use qiime composition ancombc2, which is currently in development. ANCOMBC2 models allow for repeated measurements, making it possible to run differential abundance throughout the course of the study. This would remove the need for users to filter down to their baseline and donor samples to run the comparison and will enable a more holistic comparison of indicator features throughout the study.

#### Resilience.

Resilience is an important aspect of a successful engraftment. With an FMT, researchers/clinicians are trying to replace the recipient’s microbiome with the donated microbiome, so it is important to see that the donated microbiome is stable in the recipient following FMT, rather than simply introducing a brief disturbance. Although it is common for the microbiome to drift or become personalized again after FMT (as seen in Kang et al. 2019) [5], stability of the donated microbiome is typically seen in the weeks or months following successful FMT intervention. No obvious threshold for how long the stability lasts before personalization begins has yet been identified.

q2-fmt investigates resilience through its focus on longitudinal data analysis in all actions. While all actions in q2-fmt can be run with just donated microbiome samples and recipient samples pre- and post-FMT, the actions are intended to be used with many recipient samples across time. This functionality allows researchers to understand the long term impacts of FMT on recipient microbiomes, and we hope that adoption of this tool and these approaches will facilitate an improved understanding of the temporal dynamics that should be expected following a successful FMT.

#### q2-fmt data requirements.

q2-fmt requires a minimum of three samples per recipient to investigate engraftment: a pre-FMT (baseline) sample, a post-FMT sample, and a sample of the donated material (often referred to as a “donor sample”). A donor sample may be unique to a recipient (e.g., if they are the only one who received that sample) or a single donor sample can be used with multiple recipients (e.g., if multiple recipients all received the same donor material). Additionally, samples such as pre- and post-antibiotic (or other treatments) samples and long term post-FMT samples provide a more robust analysis of engraftment. Although q2-fmt can track a single recipient’s microbial trajectory after FMT, the statistical tests like Wilcoxson SRT, Mann-Whitney-U and PEDF-permutation test do require multiple recipient subjects to assess whether engraftment measures are statistically significant.

In the sample metadata, q2-fmt requires that each sample has a metadata value indicating the sample ID of their donor. (If the recipient receives multiple doses from multiple donors, this should be annotated as donor sample ID in multiple columns - for example, columns called “initial donor sample id” and “follow-up donor sample id” can include the relevant information). Additionally, each recipient sample must have a corresponding numeric timepoint. It is helpful to collect samples from all recipients at the same timepoints to enable more consistent comparisons across recipients, but this is not strictly necessary.

## Results

**Fig 3:**
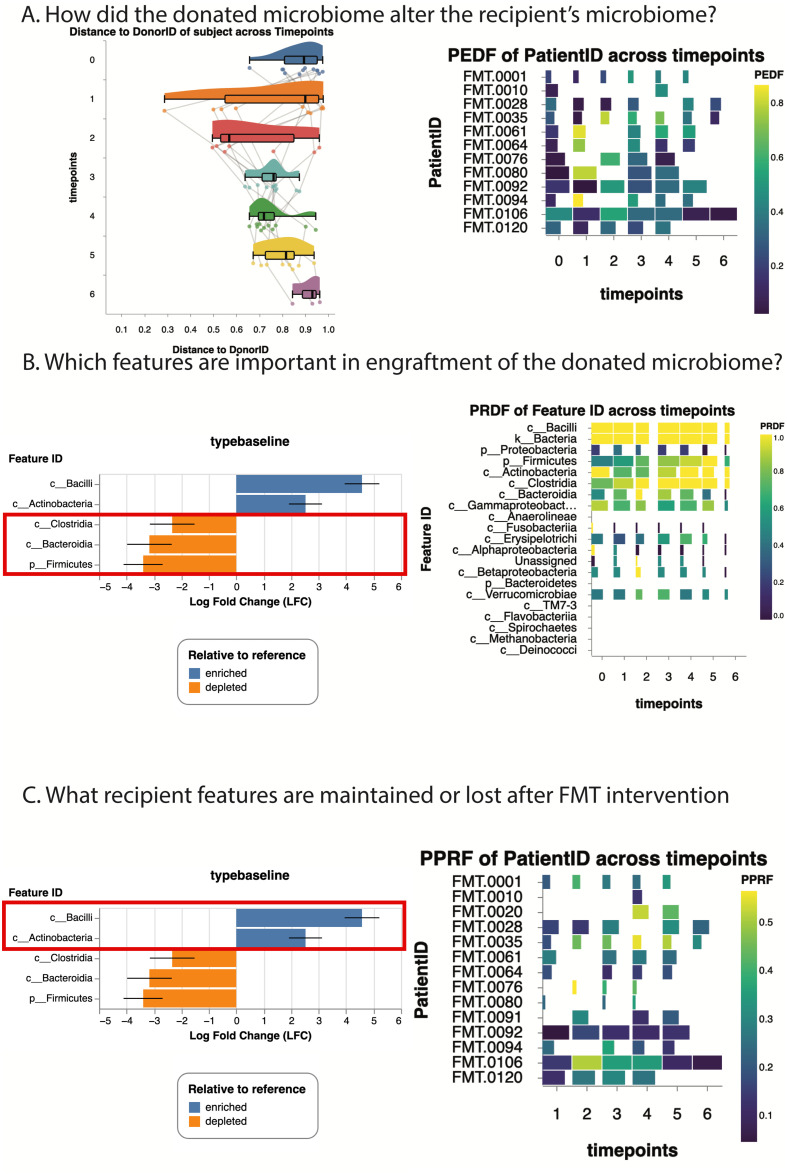
Schematic representation of possible engraftment assessments with q2-fmt. **A) How did the donated microbiome alter the recipient’s microbiome?** This subfigure illustrates that the community-coalescence pipeline and PEDF action could be used to evaluate how the donated microbiome alters the recipient’s microbiome. With longitudinal data, as presented by our tutorial data, q2-fmt also allows for investigation of the resilience of those alterations. **B) Which features are important in the engraftment?** This subfigure illustrates that the detect-donor-features pipeline and PRDF action can both assess what features are important to the engraftment, although they investigate this in different ways. The detect-donor-features pipeline evaluates which features are over-represented in the donor/reference relative to the recipient (seen in orange diverging barplots), while PRDF identifies features that successfully engrafted in multiple recipients, suggesting microbes that might be more amenable to engrafting. **C) Are recipient features maintained or lost after FMT intervention?** This subfigure illustrates the usage of detect-donor-features and PPRF to assess recipient features that are over-represented in the recipient relative to the donor/reference (seen in blue diverging barplots), and to investigate what proportion of the recipient features persist after FMT engraftment. Presented data is from Taur *et al.* (2018) [2]. Interactive versions of all figures presented here are available in the project documentation at https://q2-fmt.readthedocs.io.

q2-fmt is a comprehensive plugin providing convenient access to existing and novel methodology for assessing engraftment in six actions and two pipelines for fully assessing engraftment extent following FMT. q2-fmt provides the tools needed for any researcher to effectively assess engraftment extent in their study participants, and facilitates the use of the same metrics for tracking microbiome engraftment extent across FMT studies. q2-fmt enables FMT researchers to ask broad questions about engraftment, such as: did the donated microbiome alter the recipient microbiome (Fig 3A, CC pipeline and PEDF); what features in the donated microbiome are important in our engraftment (Fig 3B, detect-donor-features and PRDF); what recipient features are maintained or lost after FMT intervention (Fig 3C, detect-donor-features and PPRF); and is there resilience in this engraftment (by applying any of the other methods and tests to recipients with more than two timepoints) (Fig 3). q2-fmt enables standardized methodology for assessing engraftment extent following FMT, and providing insight into the most relevant microorganisms and timeframes in FMT studies.

## Availability and future directions

### Testing and maintenance

q2-fmt has a wide breadth of unit tests that cover all the actions found in the plugin. These unit tests are run on every commit to the dev branch and on every pull request.

q2-fmt can be installed in both the QIIME 2 amplicon and metagenome distributions as a community plugin using instructions in the project’s documentation. Unit tests are run weekly against the newest build of the QIIME 2 amplicon distribution and the developers are alerted to test failures. This will ensure that q2-fmt remains current with the QIIME 2 framework.

q2-fmt was developed as a part of an informatics Ph.D project at Northern Arizona University. Maintenance of this software will be performed by the Caporaso Lab at Northern Arizona University. User support for q2-fmt is provided on the QIIME 2 Forum. Worked examples of applying q2-FMT to a real-world Autologous FMT (auto-FMT) cancer microbiome study are available in the project’s documentation [2].

By nature of being implemented as a QIIME 2 plugin, q2-fmt is available through a Python 3 API, a command line interface, and through Galaxy’s graphical user interface. Analyses are fully reproducible, as a result of the QIIME 2 framework’s integrated provenance tracking and provenance replay functionality [6].

**Project name**: q2-FMT

**Project home page**: https://q2-fmt.readthedocs.io

**Operating systems**: Linux, macOS, Windows via Windows Subsystem for Linux

**Programming language**: Python 3

**Other requirements**: QIIME 2 2024.10 or later

**License**: BSD 3-Clause

**Any restrictions to use by non-academics**: None; q2-fmt is open source and free for all use.
